# An Obstructed Anomalous Left Anterior Descending Coronary Artery Arising from the Right Coronary Artery Requiring Surgical Intervention

**DOI:** 10.1155/2016/3589214

**Published:** 2016-09-19

**Authors:** Alexander Mironov, Sean Galligan, Aleksandre Kakauridze, Jonathan D. Marmur

**Affiliations:** Department of Cardiovascular Disease, SUNY Downstate Medical Center, Brooklyn, NY 11203, USA

## Abstract

A 47-year-old female presented to our hospital with symptoms of stable angina. Cardiac catheterization revealed a rare coronary artery anomaly of the left anterior descending (LAD) artery branching off the right coronary artery ostium. Furthermore, the anomalous LAD artery exhibited significant atherosclerotic obstruction. Our review of the literature found only nine such previously described cases. Due to the unique nature of coronary artery anomalies and their complications, we would like to contribute our case to the medical literature.

## 1. Introduction

Anomalies of the coronary arteries are a rare finding, with most being discovered incidentally and having no effect on quality of life. Nevertheless, a small percentage of patients who do develop symptoms are at especially great risk for cardiac complications. We would like to present a case in which a middle age female develops anginal symptoms and is found to have a rare congenital coronary artery anomaly (CCAA) of the LAD artery, requiring surgical intervention. Our subsequent discussion will highlight the epidemiology of such anomalies, their consequences, and a brief approach to management.

## 2. Case Presentation

A 47-year-old African American female presented with worsening angina over a one-week course. Her chest pain was centrally located and exacerbated by activity. At presentation, it had worsened to Canadian Cardiovascular Society Class IV and now radiating to both arms. She denied respiratory symptoms and palpitations. Her medical history was significant for menorrhagia related anemia (Hb 11.7) and a pulmonary embolism (PE) ten years earlier, attributed to oral contraceptive use. Family history was negative for heart disease or sudden cardiac death. She was a recreational smoker ten years previously and currently denies toxic habits.

**Figure 1:**
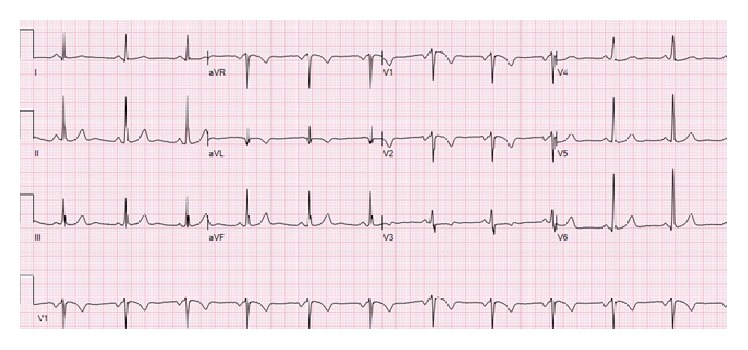
ECG 1.

The patient was evaluated for Acute Coronary Syndrome in the emergency department. ECG (see Figure 1) showed normal sinus rhythm, mild ST depressions in leads V3-V4, and T wave inversions in V1 and V2, suggestive of possible anteroseptal ischemia. Her troponin I peaked at 0.67 ng/mL and BNP was 175 pg/mL. Assessment was negative for hyperlipidemia and hyperglycemia. CT chest angiography showed no signs of PE. She was given aspirin 325 mg, clopidogrel 300 mg, and enoxaparin 1 mg/kg for NSTEMI treatment and admitted to the cardiac service.

**Figure 2:**
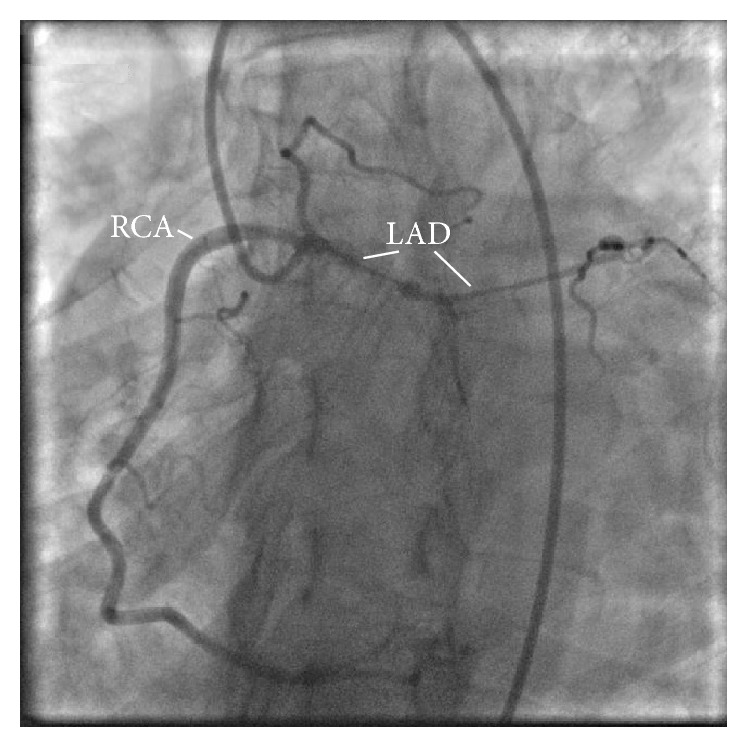
LAO caudal view of RCA. Origin of the LAD artery from the ostium of the RCA can be seen, along with severe stenosis of the proximal portion of the LAD artery.

**Figure 3:**
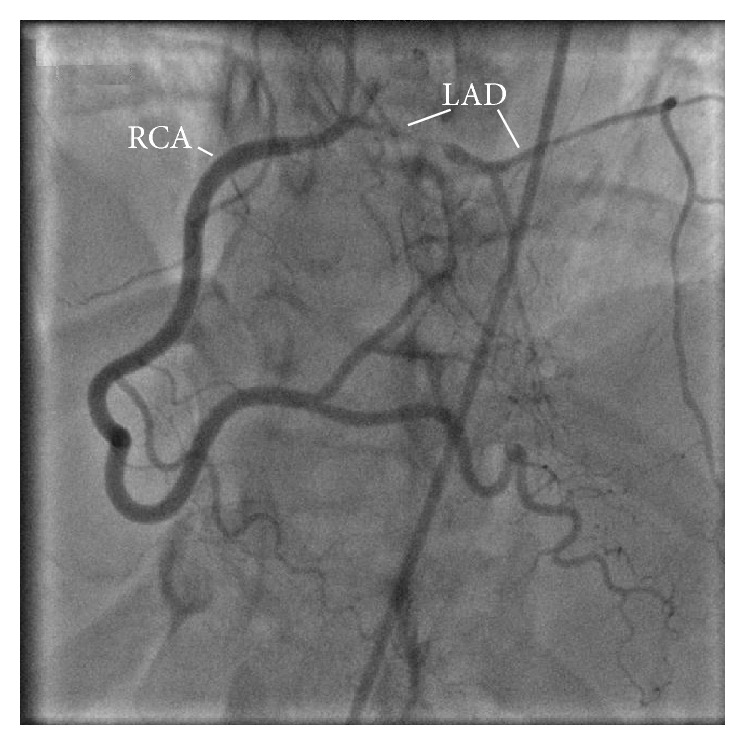
LAO cranial view of the RCA. Origin of the LAD artery from the ostium of the RCA can be seen, along with severe stenosis of the proximal portion of the LAD artery. The distal LAD artery with septal branches can also be seen.

**Figure 4:**
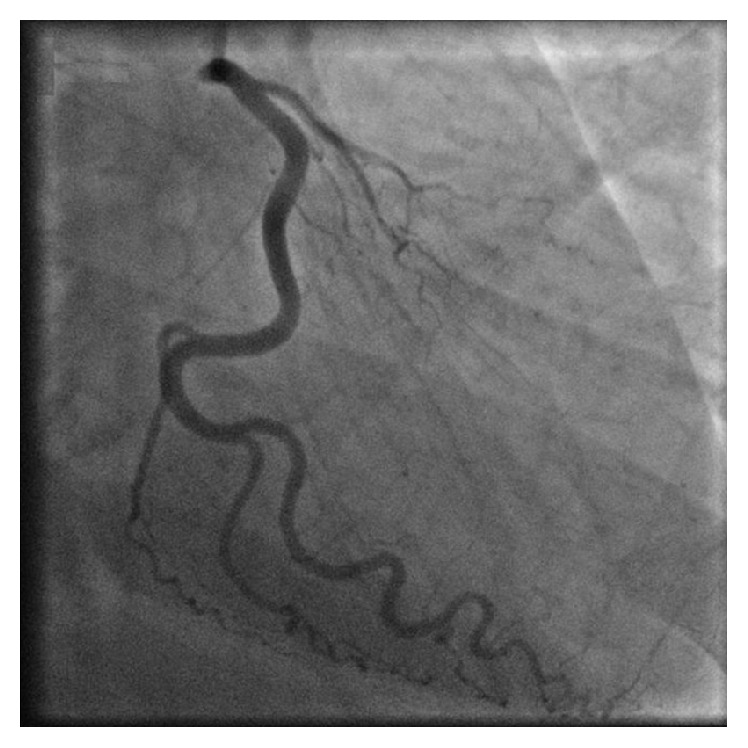
RAO caudal view of the left main artery giving rise to left circumflex artery, with no LAD artery visualized. Mild ostial 20–30% obstruction of the ostial left main artery is seen. There is a small OM1/Ramus seen which does not appear to give off septal branches.

The following morning, our patient underwent left heart cardiac catheterization which revealed single vessel disease with a 99% diffuse obstruction of the LAD artery. Furthermore, the LAD artery had a very rare anomalous origin from the ostium of the right coronary artery (RCA). The LAD artery then crossed the myocardium and descended towards the apex along the interventricular groove (see Figures 2 and 3). Meanwhile, the left coronary artery gave rise only to the circumflex artery (see Figure 4). The ostial RCA itself was noted to have a 30% obstruction. Additional findings included right dominant coronary circulation, a 20–30% ostial obstruction of the left main coronary artery, and mildly depressed left ventricle (LV) function with anterolateral hypokinesis and an ejection fraction (EF) of 45%. Lastly, the mitral valve (MV) was observed to have moderate to severe regurgitation and mild to moderate thickening of the anterior MV leaflet with no stenosis, as confirmed by transesophageal echocardiogram.

A decision was made to not pursue percutaneous coronary intervention due to potential for compromise of a very large RCA ostium. The patient was subsequently transferred to the cardiothoracic surgery service. She underwent successful left internal mammary artery to LAD artery graft and MV repair with annuloplasty ring. Postoperative course was uneventful with mechanical ventilation and vasopressor agents being discontinued on the first postoperative day. Repeat transthoracic echocardiogram (TTE) ten days after presentation demonstrated normalized LV EF of 55% and only mild MV regurgitation. After a twelve-day hospital course, our patient was discharged home in stable condition with scheduled follow-up. Her outpatient medication regimen included aspirin 325 mg daily, atenolol 50 mg twice daily, enalapril 5 mg twice daily, atorvastatin 20 mg daily, and furosemide 40 mg daily.

## 3. Discussion

Congenital coronary artery anomalies are a rare phenomenon. The incidence in the general population ranges from 0.24% to 2.33%, with the largest known study reporting 1.3% [1, 7–10]. A single anomalous LAD artery originating from the RCA, as found in our patient, is further scarce and we are aware of only nine such cases described in the literature (see Table 1) [1–6]. These cases are distinct from another anomaly which may involve an LAD artery branching off the RCA called the Type IV “Dual LAD” artery whose incidence ranges from 0.01 to 0.03% [11, 12]. In comparison, more prevalent anomalies of the LAD artery include separate origin from the circumflex artery at the left sinus of valsalva or at the right sinus of valsalva, respectively, accounting for 30.4% and 2.3% of CCAAs [7].

In addition to being able to identify CCAAs, it is important to understand their consequences. The majority are discovered incidentally and up to 81% are considered benign with no clinical significance. Meanwhile, those that are malignant have the potential to cause cardiac ischemia, especially during times of exertion and increased myocardial oxygen demand. This may lead to angina, syncope, cardiac arrhythmia, heart failure, or sudden cardiac death (SCD) [7]. In fact, coronary anomalies are the second leading cause of SCD in athletes at 19%, after hypertrophic cardiomyopathy at 36% [13].

Anatomic characteristics that make a CCAA malignant include (1) single coronary artery, (2) origin from the pulmonary artery, (3) origin from the opposite aortic sinus, (4) passing between the aorta and pulmonary artery, (5) passing intramurally, (6) acute-angle takeoff resulting in a slitlike orifice, and (7) small artery due to ostial stenosis or atresia. Furthermore, myocardial squeezing, vasospasm, and the development of atherosclerosis, as in our case, of the anomalous artery all have the potential to cause clinical symptoms [1, 3, 7, 14].

Management of anomalous arteries requires careful consideration. If a patient is identified to have a symptomatic malignant CCAA, corrective surgery is warranted. The difficulty occurs when asymptomatic patients are discovered to have a malignant CCAA. Due to the overall rarity of SCD, especially over the ages of 30–35, some may recommend avoiding surgery [15]. Meanwhile, atherosclerotic lesions may be treated with percutaneous stenting if the coronary anatomy is amenable to instrumentation [4, 5]. Otherwise, as in the case above, a bypass graft may be used. Lastly, while there is no known pattern of genetic inheritance of CCAAs, some authors have suggested screening at risk family members with TTE [16, 17].

## 4. Conclusion

While the incidence of CCAAs is low in the general population, anomalies with malignant characteristics have the potential for deadly consequences. The risks and benefits of intervention must be weighed, requiring a multidisciplinary approach with input from cardiology and cardiothoracic surgery specialists. In the case presented, an atherosclerotic anomalous LAD artery branching off the RCA combined with significant MV regurgitation necessitated a surgical approach.

## Figures and Tables

**Table 1:** Past cases of single anomalous LAD artery originating from the RCA.

Reference	Age, gender	Presenting symptom	Treatment modality	Other CCAAs present
Tuncer et al. [1]	63, F	Angina	No intervention^**∗**^	No
Tuncer et al. [1]	66, M	Angina	Bypass graft	Yes
Tuncer et al. [1]	55, M	Angina	No intervention^**∗**^	No
Nascimento et al. [2]	57, M	Angina	No intervention^**∗**^	No
Ono et al. [3]	66, F	Angina	Bypass graft	No
Ono et al. [3]	42, M	Angina	Bypass graft	No
Masuda et al. [4]	76, M	Angina	Stent	Yes
Weiguo et al. [5]	67, M	Angina	Stent	No
Russo et al. [6]	51, F	Angina	No intervention^**∗**^	No

^*∗*^Anomalous LAD artery was not the source of angina; hence, anomalous LAD artery did not require intervention and may be considered an incidental finding.
